# Cardiovascular Characteristics of Patients with Genetic Variation in Desmoplakin (*DSP*)

**DOI:** 10.3390/cardiogenetics12010003

**Published:** 2022-01-06

**Authors:** Nosheen Reza, Alejandro de Feria, Jessica L. Chowns, Lily Hoffman-Andrews, Laura Vann, Jessica Kim, Amy Marzolf, Anjali Tiku Owens

**Affiliations:** Division of Cardiovascular Medicine, Perelman School of Medicine at the University of Pennsylvania, Philadelphia, PA 19104, USA;

**Keywords:** desmoplakin, arrhythmogenic cardiomyopathy, dilated cardiomyopathy, genetics, heart failure, phenotype, genotype, arrhythmia

## Abstract

**Background:**

Variants in the desmoplakin (*DSP*) gene have been recognized in association with the pathogenesis of arrhythmogenic right ventricular cardiomyopathy (ARVC) for nearly 20 years. More recently, genetic variation in *DSP* has also been associated with left-dominant arrhythmogenic cardiomyopathy. Data regarding the cardiac phenotypes associated with genetic variation in *DSP* have been largely accumulated from phenotype-first studies of ARVC.

**Methods:**

We aimed to evaluate the clinical manifestations of cardiac disease associated with variants in *DSP* through a genotype-first approach employed in the University of Pennsylvania Center for Inherited Cardiovascular Disease registry. We performed a retrospective study of 19 individuals with “pathogenic” or “likely pathogenic” variants in *DSP* identified by clinical genetic testing. Demographics and clinical characteristics were collected.

**Results:**

Among individuals with disease-causing variants in *DSP*, nearly 40% had left ventricular enlargement at initial assessment. Malignant arrhythmias were prevalent in this cohort (42%) with a high proportion of individuals undergoing primary and secondary prevention implantable cardioverter defibrillator implantation (68%) and ablation of ventricular arrhythmias (16%). Probands also experienced end-stage heart failure requiring heart transplantation (11%).

**Conclusions:**

Our data suggest *DSP* cardiomyopathy may manifest with a high burden of heart failure and arrhythmic events, highlighting its importance in the pathogenesis of dilated and arrhythmogenic cardiomyopathies. Targeted strategies for diagnosis and risk stratification for *DSP* cardiomyopathy should be investigated.

## 1. Introduction

The expanding use of clinical genetic testing in patients with heart failure is facilitating new insights into the causes of dilated cardiomyopathy (DCM). DCM has been defined by the presence of: (a) fractional shortening less than 25% (>2 SD) and/or ejection fraction less than 45% (>2 SD) and (b) left ventricular end diastolic diameter greater than 117% (>2 SD of the predicted value of 112% corrected for age and body surface area), in the absence of another myocardial, valvular, or systemic etiology of cardiomyopathy [1]. Familial DCM, a subtype of DCM, is suspected when (a) two or more affected relatives with DCM meet the previously mentioned criteria or (b) a relative of a DCM patient experienced unexplained sudden death before the age of 35 years [2]. The prevalence of familial DCM is estimated to be 30–50% [1,2]. Forty percent of familial DCM has an identifiable genetic cause and over 60 genes associated with familial DCM have been reported [1].

Natural history studies have demonstrated that pathogenic variants in DCM-associated genes, such as lamin A/C (*LMNA*), filamin C (*FLNC*), cardiac sodium channel NAv1.5 (*SCN5A*), and RNA binding motif protein 20 (*RBM20*), lead to a malignant arrhythmogenic phenotype, which can be unrelated to the degree of left ventricular (LV) dysfunction [1]. Arrhythmogenic DCM, which has been found in one-third of DCM patients [3], has emerged as an overlap phenotype with arrhythmogenic cardiomyopathy (ACM) and arrhythmogenic left/left-dominant arrhythmogenic cardiomyopathy (ALVC) [4]. The lack of specific and widely accepted diagnostic criteria for ACM and ALVC has limited the recognition and categorization of these entities.

The desmosome and its components regulate cell–cell communication. Desmoplakin (*DSP*), a desmosomal protein essential to cardiac force transmission, functions as a linker protein and connects the desmosome to a network of cytoskeletal proteins, including intermediate filament proteins [5–8]. Disruption in these desmosomal structural components can result in myocardial structural and electrical alterations. Recently, the ACM/ALVC phenotype has been identified in association with genetic variation in desmin (*DES*), the gene that encodes a multipurpose intermediate filament protein [9,10].

**Figure 1.**
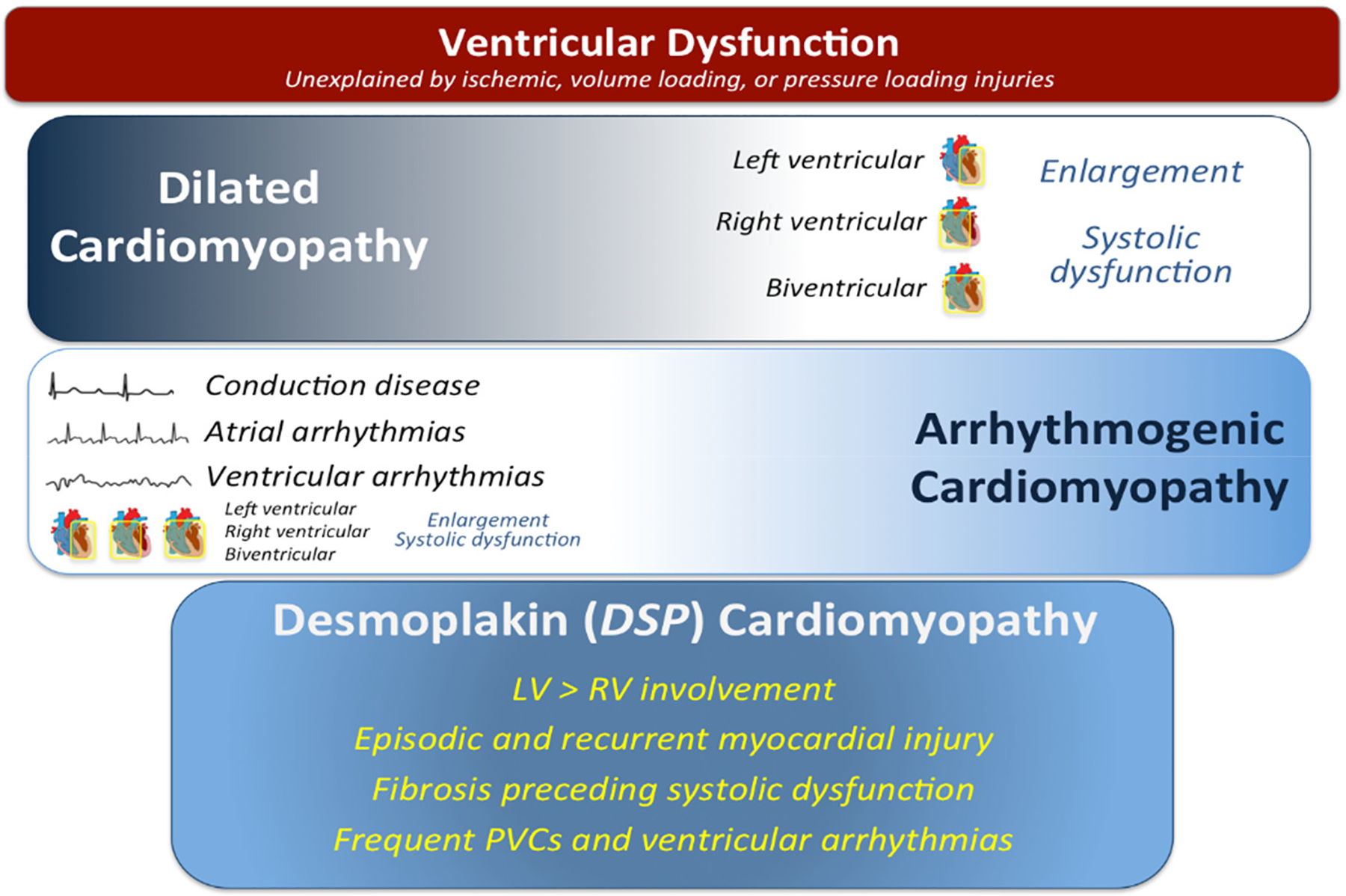
Characteristics of the clinical presentations of dilated cardiomyopathy (DCM), arrhythmogenic cardiomyopathy (ACM), and desmoplakin cardiomyopathy. Dilated cardiomyopathy is typically characterized by ventricular systolic dilation and dysfunction in the absence an ischemic, valvular, hypertensive, or other systemic insult. Arrhythmogenic cardiomyopathy is distinguished by a clinical presentation with documented or symptomatic arrhythmia or conduction disease. This presentation can occur concomitantly with ventricular dilation and/or dysfunction. Desmoplakin cardiomyopathy presents with features of both DCM and ACM along with unique features such as episodic and recurrent myocardial injury. Abbreviations: DSP = desmoplakin; LV = left ventricular; RV = right ventricular; PVCs = premature ventricular contractions.

Genetic variation in *DSP* has been causally implicated in arrhythmogenic right ventricular cardiomyopathy (ARVC), with more recent suspicion in the pathogenesis of ALVC. A majority of the investigation of *DSP*-associated disease has been performed in cohorts of individuals with ARVC [11–18]. Emerging data from ACM cohorts have suggested features of *DSP*-associated cardiac disease that may be distinct from DCM, ARVC, and ALVC (Figure 1). We hypothesize that the predominantly right ventricular phenotype-first approach utilized to date to investigate *DSP*-associated cardiac disease has limited the identification of important clinical features and outcomes. In this study, we aimed to conduct a genotype-first approach to investigate the clinical manifestations of genetic variation in *DSP*.

## 2. Methods

### 2.1. Subjects and Study Design

Individuals evaluated at the University of Pennsylvania Center for Inherited Cardiovascular Disease, a tertiary referral center for patients with suspected hereditary cardiomyopathies and otherwise unexplained heart muscle diseases, from 2011–2019 were eligible for inclusion. Individuals with “pathogenic” or “likely pathogenic” variants in *DSP* identified by Clinical Laboratory Improvement Amendments-certified clinical genetic testing and consistent with published standards by the American College of Medical Genetics and Genomics and the Association for Molecular Pathology were included [19]. Cascade screening of first-degree relatives for all individuals with cardiomyopathy was routinely recommended as per current practice guidelines [20]. Probands and first-degree family members were classified as such. Clinical genetic testing results were independently confirmed by licensed genetic counselors (J.C., L.H.-A.). Entry into the cohort was assigned by date of initial evaluation by a cardiologist in the University of Pennsylvania Health System. The Institutional Review Board of the University of Pennsylvania approved this study (protocol number 843087). Due to the retrospective nature of the study and waiver granted by the Institutional Review Board, no informed consent from the subjects was required.

### 2.2. Data Collection

Data obtained by review of medical records included clinical history, pedigree analysis, electrocardiography (ECG), transthoracic echocardiography (TTE), ambulatory electrocardiographic monitoring (MCOT), cardiac magnetic resonance imaging (CMR), treatment, and follow-up testing as clinically indicated. Baseline demographic data included age at diagnosis, sex, first-degree familial history of DCM or sudden cardiac death, and symptoms at initial visit. Testing was performed according to standard clinical protocols. Echocardiographic chamber quantification was performed in alignment with the 2015 American Society of Echocardiography/European Association of Cardiovascular Imaging recommendations on cardiac chamber quantification for adults [21]. Clinical outcomes were adjudicated by medical record review.

### 2.3. Statistical Analysis

Continuous variables were described with medians and interquartile ranges (IQR) and categorical variables as n (%) with group comparisons performed with Wilcoxon rank sum testing. Kaplan–Meier curves were produced to analyze event-free probability from date of initial evaluation to a composite event (ventricular arrhythmia ablation, heart transplantation) with right censoring at date of death or date of last follow-up and were compared using the log-rank test. A 2-sided *p* value < 0.05 was considered statistically significant. Analyses were performed with Stata 15.1/IC (College Station, TX, USA).

## 3. Results

### 3.1. Clinical Presentation of Probands and Relatives

Between 2011 and 2019, a total of 19 patients, 11 probands and 8 relatives, from 14 different families were identified (Table 1). Data on variant type and pathogenicity are presented in Table 2. Of 14 unique variants found, 7 had not been previously cited in ClinVar, the United States National Institutes of Health public archive of human genetic variants (as of 20 March 2021) [22]. Age at diagnosis was not significantly different between relatives (median 35.5 years, IQR 31–48) and probands (42 years, IQR 30–48; *p* = 0.53). Five probands (26%) had a family history of sudden cardiac death, and five probands had a family history of DCM. At initial evaluation, most probands and relatives were asymptomatic or mildly symptomatic by New York Heart Association (NYHA) class. Palpitations were the most common symptom reported by both probands (27%) and relatives (63%) at initial evaluation.

### 3.2. Electrocardiography and Arrhythmias

Eight of eleven probands (73%) and six of eight relatives (75%) had abnormal 12-lead ECGs on initial electrocardiographic screening. Over half of the probands (55%) and 25% of relatives had T wave inversions in leads II, III, and aVF. T wave inversions in the lateral precordial leads (V4, V5, V6) were more frequent among probands compared with T wave inversions in leads V1, V2, and V3 (*p* < 0.01).

All 11 probands and 6 relatives (75%) experienced either or both atrial and ventricular arrhythmias. Probands had a significantly higher burden of ventricular ectopy as compared to relatives (median 554.1 beats per hour (IQR 194.3–1024.3) vs. 5.9 (IQR 1.5–103.4); *p* = 0.02). Nonsustained ventricular tachycardia (VT) was common in both groups but occurred significantly more frequently among probands compared to relatives (*p* = 0.02). Five probands (45%) and one relative (13%) experienced sustained VT. Atrial fibrillation was rare and occurred in one proband (9%) and one relative (13%).

Nine probands (81%) had implantable cardioverter defibrillators (ICD), six of which were implanted for primary prevention. Median LVEF at the time of ICD implantation in the probands was 36% (IQR 28–47). Three probands received appropriate ICD shocks over a median 62.8 months of follow-up (IQR 32.8–173.5) and none received an inappropriate shock. Five probands (45%) underwent catheter-directed ablation of either premature ventricular contractions (PVC) or VT.

Four relatives (50%) had an ICD, three of which were for primary prevention. Median LVEF at the time of ICD implantation in the 4 relatives was 32% (IQR 28–45). No relatives received appropriate ICD shocks, and one received an inappropriate shock for sinus tachycardia over a median 20.9 months of follow-up (IQR 13.7–37.0). For the three individuals with documented LVEF at the time of secondary prevention ICD implantation, LVEFs were 49%, 55%, and 60%.

Median LVEF at the time of ICD implantation was significantly higher for those who underwent secondary prevention ICD versus primary prevention (55% vs. 30%, *p* = 0.01). Of the seven individuals who met 2010 ARVC Task Force criteria for “definite” ARVC [23], three had no phenotypic evidence of RV disease and four had evidence of biventricular cardiomyopathy (Table 3).

### 3.3. Cardiovascular Imaging

Left ventricular ejection fraction (LVEF) was higher for relatives (46%, IQR 25–63) at first contact compared to probands (30%, IQR 25–45), though this difference was not statistically significant (*p* = 0.59). Four probands (36%) and three relatives (38%) had left ventricular enlargement by TTE at the time of first contact. Left ventricular end diastolic diameter (LVEDD) was larger for probands (57 mm, IQR 50–58) at first contact compared to relatives (48.5 mm, IQR 45–57), though this difference was not statistically significant (*p* = 0.13). LVEF and LVEDD did not significantly change for either probands or relatives over a median follow-up of 36.3 months (IQR 9.9–72.5). At the time of most recent follow-up, most probands and relatives had normal RV size (82% and 63%, respectively) and normal RV function (82% and 75%, respectively) on TTE.

**Figure 2.**
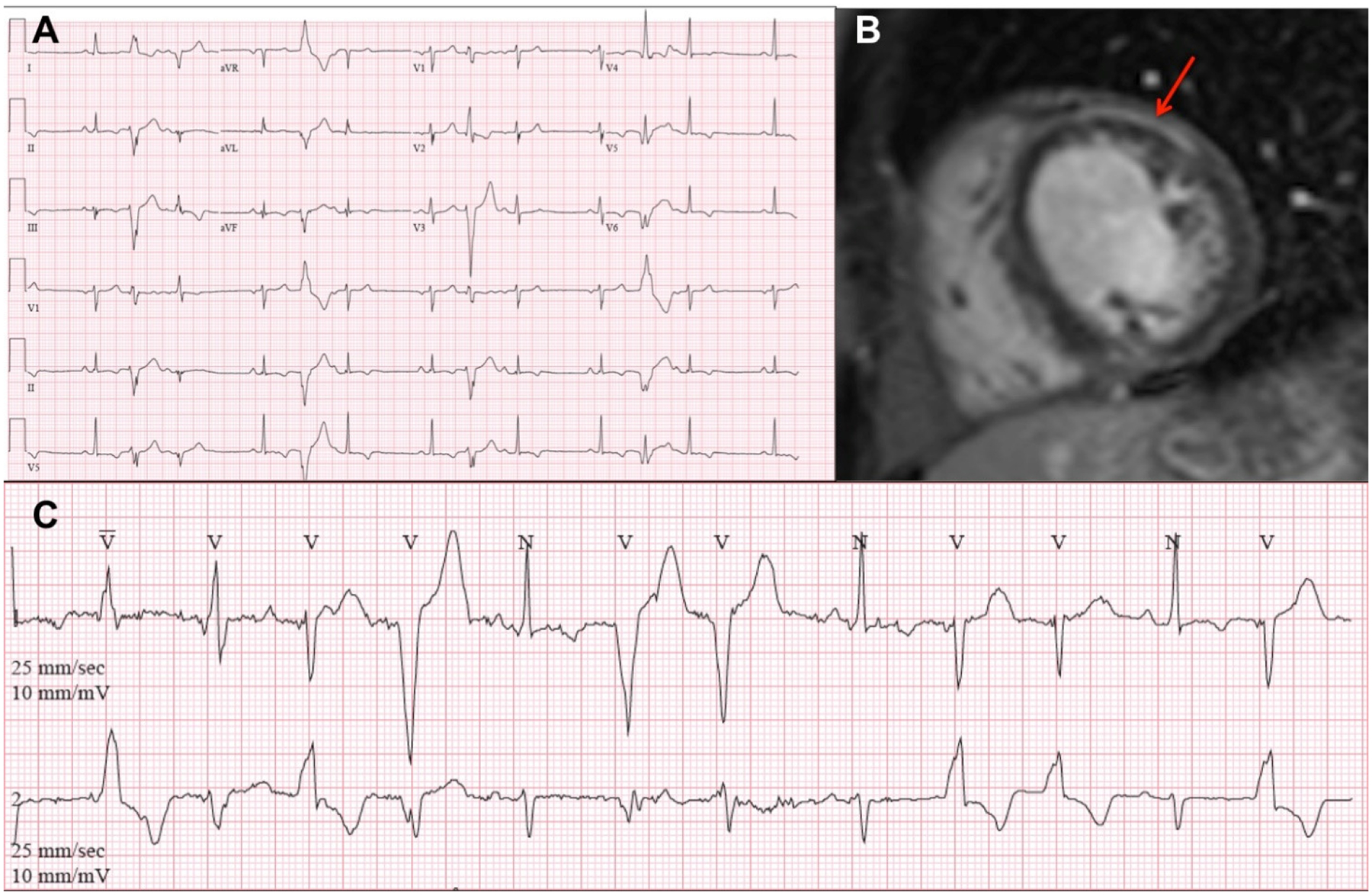
Electrocardiography and cardiac magnetic resonance imaging of two patients with pathogenic truncating variants in the desmoplakin (*DSP*) gene. 12-lead (**A**) and extended outpatient electrocardiographic monitoring (**C**) of Patient ID 13 (c. 888C > G, p. Tyr296*) demonstrated frequent multifocal premature ventricular complexes. Paper speed and amplification: 25 mm/s and 1 mV/10 mm. Cardiac magnetic resonance imaging (**B**) with contrast of Patient 15 (c. 478C > T, p. Arg160*) demonstrated subepicardial late gadolinium enhancement in the left ventricular anterolateral wall at the mid-cavity (arrow).

Thirteen patients underwent CMR either at our or an outside institution. Twelve patients (92%) had LV late gadolinium enhancement (LGE), while only one proband and one relative (unrelated) had RV LGE (Table 4). Among probands, LV LGE occurred most commonly in the subepicardial and mid-myocardial layers (Figure 2).

### 3.4. Histopathology

Myocardial histopathology was available from 5 of 19 patients: right ventricle (Patient 1), left ventricle (Patients 15 and 19), and native explanted heart (Patients 7 and 9). All samples demonstrated myocyte hypertrophy and mild to moderate endocardial and interstitial fibrosis. No sample demonstrated granulomas, giant cells, significant inflammatory infiltrates, amyloid, or iron deposition. Periodic acid–Schiff staining with and without diastase on Patient 15′s sample revealed presence of intracellular glycogen; however, this was not demonstrated on other LV samples. Histopathology findings are summarized in Table 5.

### 3.5. Outcomes

**Figure 3.**
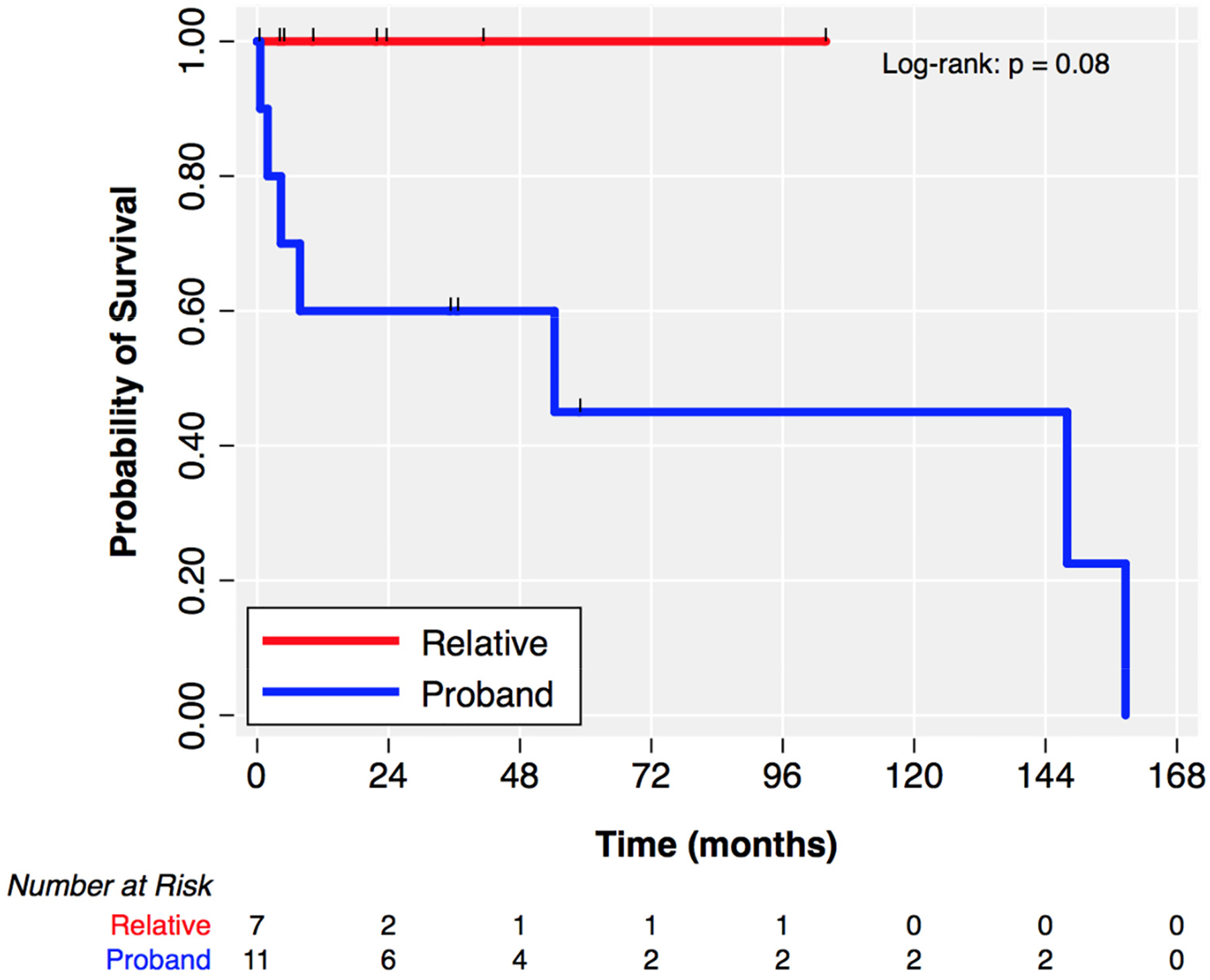
Kaplan–Meier estimates of event-free survival for the composite outcome of ventricular arrhythmia ablation or heart transplantation with right censoring at date of death or date of last follow-up. Tick marks indicate censored individuals. Over the observed time of follow-up, no relative underwent ventricular arrhythmia ablation or heart transplantation.

Over a median follow-up of 36.3 months (IQR 9.9–72.5), one relative required extracorporeal membrane oxygenation for cardiogenic shock and eventually died. Two probands underwent heart transplantation for NYHA IV/Stage D heart failure and refractory ventricular arrhythmias (Table 6). Kaplan–Meier curves showing a comparison of the incidence of the composite outcome of PVC ablation, VT ablation, and heart transplantation for probands and relatives are shown in Figure 3.

## 4. Discussion

Current knowledge regarding clinical phenotypes and outcomes of desmoplakin cardiomyopathies is largely based on published reports of small populations [5], including (1) large cohorts of individuals with ARVC [11–13,15,17,18,24], (2) other cardio-myopathy cohorts with fewer than 10 probands [25–28], and (3) single family/single *DSP* variant cohorts [29,30]. Our study is one of the larger single-center descriptions of the clinical characteristics of *DSP* cardiomyopathy and aligns with recent work describing the distinctive presentation and course of this disease and the importance of molecular diagnosis for appropriate detection and risk stratification [8,31].

In this cohort of 19 individuals with pathogenic or likely pathogenic non-missense *DSP* variants, we found a high prevalence of left ventricular pathology by TTE and CMR. Probands and relatives exhibited LV systolic dysfunction relatively early, in the third to fourth decades of life, and similar to the average age at diagnoses of other genetic ACM, such as LMNA-associated DCM. Right ventricular dilation and dysfunction by TTE and/or CMR were rare in this cohort, supporting emerging knowledge that there may be a subset of individuals with *DSP* cardiomyopathy who do not manifest with the classical ARVC phenotype. The possibility of a distinct pathogenesis of *DSP*-associated disease, compared to other desmosomal cardiomyopathies, has been recently suggested [32,33]. Only 7 of 19 individuals met the 2010 Task Force criteria for a “definite” diagnosis of ARVC, despite each individual having one major criterion fulfilled at baseline with the presence of a pathogenic/likely pathogenic *DSP* variant. Our findings align with a recent multicenter study from Smith et al. that similarly highlighted the poor sensitivity of the 2010 Task Force criteria in identifying individuals with *DSP* cardiomyopathy [8]. The high prevalence of LV LGE on CMR in our cohort indicates that LV scarring and fibrosis, particularly in the subepicardial and mid-myocardial layers, might warrant consideration as a characteristic injury pattern in *DSP* cardiomyopathy. The etiology of this scarring and fibrosis remains unclear; however, autoimmunity and myocarditis have been proposed [34,35]. Further efforts to link biomarkers, histopathology, and imaging longitudinally in individuals with *DSP* cardiomyopathy will be critical to our understanding of the etiology and sequelae of the desmosomal cardiomyopathies.

We describe a high prevalence of atrial and ventricular arrhythmias and sudden cardiac arrest in this cohort. In addition, we report substantial utilization of therapies related to the treatment of malignant ventricular arrhythmias and heart failure including, secondary prevention ICD implantation, appropriate ICD shocks, ventricular arrhythmia ablation, and heart transplantation. Our observations support recent work from Wang et al., derived from a single center prospective registry of ARVC [31], which demonstrated that individuals with disease-causing variants in *DSP* were at high risk for sustained ventricular arrhythmia and heart failure. Importantly, the three individuals in our cohort who underwent secondary prevention ICD implantation had LVEFs above the guideline recommended primary prevention ICD implantation threshold of <35%. Although a small sample, this raises the question of using LVEF thresholds established for DCM in *DSP* cardiomyopathy, similar to the exception recommended for LMNA cardiomyopathy [4]. Our findings also add further evidence that left-sided and biventricular involvement, and not just right ventricular disease, should be suspected in *DSP* cardiomyopathies.

Limitations of our study include its single center retrospective nature with a small sample size, resulting in limited direct genotype-phenotype associations, possible referral bias, and possible lead time bias with differences between probands and relatives. Complete clinical data, including dermatologic phenotyping, was not available for all individuals. We acknowledge that the *TNNT2* variant found in Patient ID 7 has been reported in association with DCM and may have influenced this individual’s phenotype. In frame deletions in *DMD* are associated with the X-linked recessive Becker muscular dystrophy, and this female patient (Patient ID 13) did not exhibit signs or symptoms of skeletal myopathy. Digenic heterozygosity in desmosomal genes has been reported as a potential contributor to the development of an ARVC phenotype [36–39]; however, the characteristics of individuals with digenic heterozygosity in *DSP* and either *TNNT2* or *DMD* has not been previously reported.

## 5. Conclusions

Variants in *DSP* are estimated to occur in 3% of European individuals with DCM; although they have been historically implicated in ARVC, we demonstrate that disease-causing non-missense *DSP* variants manifest with a high burden of LV injury, LV dysfunction, heart failure, and arrhythmic events. We highlight the distinct features of *DSP* cardiomyopathy and association of *DSP* in the pathogenesis of DCM and ALVC, supporting the need to incorporate a genotype-first approach and molecular diagnostics into risk assessment and clinical care.

## Figures and Tables

**Table 1.** Demographics and clinical characteristics of 19 probands and relatives with pathogenic or likely pathogenic variants in the desmoplakin gene (*DSP*).

Characteristic	Relatives	Probands	*p*-Value
	N = 8	N = 11	
**Age at Diagnosis (years), median (IQR)**	35.5 (31.0–48.0)	42.0 (30.0–48.0)	0.53
**Sex (%)**			1.00
Female	5 (62%)	7 (64%)	
Male	3 (38%)	4 (36%)	
**Race/Ethnicity (%)**			1.00
Hispanic Latinx/White	1 (12%)	1 (9%)	
White	7 (88%)	9 (82%)	
Unknown	0 (0%)	1 (9%)	
**Symptoms at Initial Assessment (%)**			
NYHA Class			0.11
I	5 (62%)	6 (55%)	
II	1 (12%)	5 (45%)	
III	2 (25%)	0 (0%)	
Dyspnea	2 (25%)	1 (9%)	0.55
Chest Pain	2 (25%)	1 (9%)	0.55
Palpitations	5 (62%)	3 (27%)	0.18
Presyncope	1 (12%)	2 (18%)	1.00
Syncope	1 (12%)	0 (0%)	0.42
Edema	0 (0%)	0 (0%)	1.00
**Family History of SCD (%)**	—	5 (45%)	
**Family History of DCM (%)**	—	5 (45%)	

Abbreviations: NYHA = New York Heart Association; SCD = sudden cardiac death; DCM = dilated cardiomyopathy.

**Table 2.** Pathogenic and likely pathogenic variants in the desmoplakin gene (*DSP*) in 19 patients.

Patient ID	Family Number	Nucleotide Change (c.)	Amino Acid Change (p.)	Variant Type	ClinVar Citations	ACMG Classification
1	1	5940dupC	Tyr1981fs	Frameshift	None	Likely pathogenic
2	2	6767delG	Gly2256Valfs*5	Frameshift	None	Pathogenic
3	2	6767delG	Gly2256Valfs*5	Frameshift	None	Pathogenic
4	3	5212C > T	Arg1738Ter	Nonsense	PMID: 28492532	Pathogenic
					PMID: 26314686	
					PMID: 29759408	
					PMID: 25616645	
5	4	939 + 1G > A	IVS7 + 1 G > A	Splice site	PMID: 24503780	Pathogenic
					PMID: 20864495	
					PMID: 19558499	
					PMID: 19279339	
					PMID: 19095136	
					PMID: 10594734	
6	5	7491_7492delTG	Cys2497Ter	Truncating	None	Likely pathogenic
7 *	5	7491_7492delTG	Cys2497Ter	Truncating	None	Likely pathogenic
8	6	3799C > T	Arg1267Ter	Truncating	PMID: 16467215	Likely pathogenic
9	7	4999C > T	Gln1667Ter	Nonsense	PMID: 28492532	Pathogenic
10	7	4999C > T	Gln1667Ter	Nonsense	PMID: 28492532	Pathogenic
11	7	4999C > T	Gln1667Ter	Nonsense	PMID: 28492532	Pathogenic
12	8	5851C > T	Arg1951Ter	Truncating	PMID: 28527814	Pathogenic
					PMID: 26899768	
					PMID: 21859740	
					PMID: 11063735	
13 *	9	888C > G	Tyr296*	Truncating	None	Pathogenic
14	10	313C > T	Arg105*	Truncating	None	Pathogenic
15	11	478C > T	Arg160*	Truncating	PMID: 28588093	Pathogenic
					PMID: 28492532	
					PMID: 27532257	
					PMID: 26850880	
					PMID: 25616645	
					PMID: 23810894	
16	12	3415_3417delinsG	Tyr1139Glyfs*10	Truncating	None	Pathogenic
17	7	4999C > T	Gln1667Ter	Nonsense	PMID: 28492532	Pathogenic
18	13	7372_7373delAA	Lys2458GlufsX7	Truncating	None	Likely pathogenic
19	14	4531C > T	Gln1511*	Truncating	PMID: 28492532	Pathogenic

* Patient ID 7 also carried a pathogenic truncating variant (c.629_631delAGA, p.Lys210del) in *TNNT2* (cardiac troponin T). Patient ID 13 also carried an in-frame deletion (exons 49–51) in *DMD* (dystrophin). Classifications were based on interpretations from Clinical Laboratory Improvement Amendments-certified laboratories and confirmed by institutional genetic counselors.

Abbreviations: ID = identifier; ACMG = American College of Medical Genetics and Genomics.

**Table 3.** Electrocardiographic and arrhythmia characteristics of 19 probands and relatives with pathogenic or likely pathogenic variants in the desmoplakin gene (*DSP*).

Characteristic	Relatives	Probands	*p*-Value
	N = 8	N = 11	
**T wave inversions, II, III, aVF**	2 (25%)	6 (55%)	0.35
**T wave inversions, V1–V2**	3 (38%)	0 (0%)	0.06
**T wave inversions, V1–V3**	3 (38%)	0 (0%)	0.06
**T wave inversions, V3–V4**	2 (25%)	2 (18%)	1.00
**T wave inversions, V4–V6**	3 (38%)	3 (27%)	1.00
**T wave inversions, V5–V6**	4 (50%)	5 (45%)	1.00
**Ventricular ectopy (VE) (%)**	5 (62%)	11 (100%)	0.058
**MCOT VE count, median (IQR)**	933.0 (74.0–3235.5)	16,460.5 (9319.5–35,926.0)	0.011
**MCOT hours, median (IQR)**	91.5 (36.0–165.5)	35.5 (24.0–49.0)	0.33
**MCOT VE burden (beats per hour), median (IQR)**	5.9 (1.5–103.4)	554.1 (194.3–1024.3)	0.017
**Arrhythmia (%)**	6 (75%)	11 (100%)	0.16
**NSVT (%)**	4 (50%)	11 (100%)	0.018
**VT (%)**	1 (12%)	5 (45%)	0.18
**Atrial fibrillation (%)**	1 (12%)	1 (9%)	1.00
**Supraventricular tachycardia (%)**	4 (50%)	1 (9%)	0.11
**Sudden cardiac arrest (%)**	1 (12%)	1 (9%)	1.00
**ICD (%)**	4 (50%)	9 (82%)	0.32
Primary prevention ICD	3 (38%)	6 (55%)	0.48
Secondary prevention ICD	1 (12%)	3 (27%)	0.48
**Type of ICD (%)**			0.92
Single chamber	1 (12%)	3 (27%)	
Dual chamber	1 (12%)	2 (18%)	
Subcutaneous	2 (25%)	3 (27%)	
Unknown	4 (50%)	3 (27%)	
**LVEF (%) at time of ICD implantation, median (IQR)**	32.0 (27.5–44.5)	36.0 (27.5–47.0)	0.80
**Appropriate ICD shock (%)**	0 (0%)	3 (27%)	0.21
**Inappropriate ICD shock (%)**	1 (12%)	0 (0%)	0.084
**VT catheter ablation (%)**	0 (0%)	2 (18%)	0.49
**PVC catheter ablation (%)**	0 (0%)	3 (27%)	0.23

Abbreviations: MCOT = mobile cardiac outpatient telemetry; VE = ventricular ectopy; NSVT = nonsustained ventricular tachycardia; VT = ventricular tachycardia; ICD = implantable cardioverter defibrillator; LVEF = left ventricular ejection fraction; PVC = premature ventricular contractions.

**Table 4.** Cardiovascular imaging characteristics of 19 probands and relatives with pathogenic or likely pathogenic variants in the desmoplakin gene (*DSP*).

Characteristic	Relatives	Probands	*p*-Value
	N = 8	N = 11	
**LVEF (%) by TTE at initial assessment, median (IQR)**	46.0 (24.5–62.5)	30.0 (25.0–45.0)	0.59
**LVEDD (millimeters) by TTE at initial assessment, median (IQR)**	48.5 (45.0–56.5)	57.0 (50.0–68.0)	0.13
**RV function on TTE at initial assessment (%)**			0.37
Normal	6 (75%)	6 (55%)	
Normal to Mildly Decreased	1 (12%)	0 (0%)	
Mildly Decreased	0 (0%)	1 (9%)	
Unknown	1 (12%)	4 (36%)	
**LV LGE location on CMR (%)**			0.15
Subepicardial	1 (12%)	0 (0%)	
Subepicardial + mid-myocardial	0 (0%)	4 (36%)	
Mid-myocardial	1 (12%)	2 (18%)	
Transmural	0 (0%)	1 (9%)	
Epicardial + transmural + mid myocardial	0 (0%)	1 (9%)	
Unknown	1 (12%)	1 (9%)	
No CMR	5 (62%)	2 (18%)	

Abbreviations: TTE = two-dimensional transthoracic echocardiography; LVEDD = left ventricular end diastolic diameter; RV = right ventricular; LV = left ventricular; LGE = late gadolinium enhancement; CMR = cardiac magnetic resonance imaging.

**Table 5.** Myocardial histopathology findings of five patients with pathogenic or likely pathogenic variants in the desmoplakin gene (*DSP*).

Patient ID	Specimen Type	Myocyte Hypertrophy	Fibrosis	Other Notable Findings
1	RV	Nonspecific	Mild	Increased number of mitochondria on electron microscopy
7	Explanted heart	—	Interstitial & subendocardial	No amyloid, parenchymal iron or excess glycogen deposition, granulomas, giant cells or inflammatory infiltrates
9	Explanted heart	Mild to moderate	Mild interstitial & subendocardial	No amyloid
15	LV	Severe	Interstitial	Intracellular glycogen present on PAS with and without diastase No amyloid, parenchymal iron deposition, granulomas, giant cells or inflammatory infiltrates
19	LV	Moderate	Patchy interstitial & subendocardial	—

Abbreviations: ID = identifier; RV = right ventricle; LV = left ventricle; PAS = Periodic acid–Schiff.

**Table 6.** Clinical outcomes of 19 probands and relatives with pathogenic or likely pathogenic variants in the desmoplakin gene (*DSP*).

	Relatives	Probands	*p*-Value
**N**	8	11	
**ECMO (%)**	1 (12%)	0 (0%)	0.42
**Heart Transplantation (%)**	0 (0%)	2 (18%)	0.49
**Death (%)**	1 (12%)	0 (0%)	0.42

Abbreviations: ECMO = extracorporeal membrane oxygenation.

## Data Availability

The data presented in this study are available on reasonable request from the corresponding author and are not publicly available due to the potential to compromise the privacy of research participants.
